# Luteolin: a natural product with multiple mechanisms for atherosclerosis

**DOI:** 10.3389/fphar.2025.1503832

**Published:** 2025-03-27

**Authors:** Chanjun Wan, Qingzhi Liang, Yirong Ma, Yang Wang, Liqiang Sun, Junyu Lai, Jianguang Wu, Zhengtao Chen

**Affiliations:** ^1^ Affiliated Hospital of Jiangxi University of Chinese Medicine, Nanchang, Jiangxi, China; ^2^ Jiangxi Province Key Laboratory of Cardiovascular Diseases of Chinese Medicine, Nanchang, Jiangxi, China; ^3^ Hospital of Chengdu University of Traditional Chinese Medicine, Chengdu, Sichuan, China; ^4^ Jiangxi University of Chinese Medicine, Nanchang, Jiangxi, China

**Keywords:** atherosclerosis, luteolin, pharmacological mechanisms, psychological risk factors, blood vessel cells

## Abstract

Atherosclerosis (AS) is a degenerative and proliferative disease characterised by the deposition of lipid and/or fibrous substances within the intima of arteries. The pathological mechanisms of AS involve endothelial cell (EC) injury and dysfunction, vascular smooth muscle cell (VSMC) migration and proliferation, foam cell formation, inflammatory cell recruitment, and abnormal platelet activation and aggregation. In recent years, the incidence and mortality rates of atherosclerotic cardiovascular disease (ASCVD), which has AS as its pathological basis, have shown an upward trend globally. Currently, available therapeutic agents (such as statins, PCSK9 inhibitors, and antiplatelet drugs) can, to some extent, delay the progression of AS; however, many of these drugs have adverse effects or are not suitable for long-term use, potentially causing severe negative impacts on patients’ lives and work. Therefore, the development of safe and effective therapeutic drugs holds immense social and economic significance. In recent years, natural compounds derived from plants have gradually emerged as a source of new drugs for treating AS. Luteolin (3′,4′,5,7-tetrahydroxyflavone) is a common plant-derived flavonoid widely found in various vegetables and fruits, including celery, parsley, broccoli, onion leaves, carrots, peppers, cabbage, apples, and chrysanthemums. Numerous preclinical studies have revealed that luteolin exhibits significant anti-AS effects. This article comprehensively reviews the effects of Lu on vascular cells (endothelial cells, vascular smooth muscle cells, macrophages, neutrophils) under experimental AS conditions and its regulatory effects on common AS risk factors (hypertension, hyperglycemia, dyslipidemia), providing a strong evidential basis for the clinical application and mechanistic research of luteolin.

## 1 Introduction

Atherosclerosis (As) is a complex pathological process characterised by the deposition of lipids within the arterial wall. As lipid plaques progress, the arterial wall gradually hardens and the arterial lumen subsequently becomes narrowed. These AS plaques may rupture or be eroded, triggering a series of severe clinical events such as myocardial infarction, ischaemic cardiomyopathy, stroke, and peripheral arterial occlusive disease, among other cardiovascular diseases (CVDs). The pathogenic mechanisms of AS encompass inflammation, lipid infiltration, oxidative stress, platelet hyperfunction, immune dysfunction, and shear stress (Mury et al., 2018), involving multiple pathological processes including endothelial cell injury, macrophage inflammation, foam cell formation, and vascular smooth muscle cell proliferation and migration (Libby et al., 2011). According to data from the World Health Organization (WHO), approximately 17.9 million people globally die from CVDs each year, accounting for 30% of all deaths worldwide. By 2030, this figure is projected to exceed 23.6 million. Therefore, effective prevention and treatment of AS constitute both an urgent medical challenge and a current research hotspot. However, effective means to cure or reverse AS are still lacking (Vergallo and Crea, 2020).

Current prevention and treatment methods primarily involve controlling cardiovascular risk factors (such as lipid regulation, blood pressure reduction, glycemic control, weight loss, and smoking cessation), combined with antiplatelet, thrombolytic, and anticoagulant therapies based on patients’ clinical symptoms. Surgical treatments include balloon angioplasty, stent placement, and bypass surgery. While these therapeutic approaches can, to some extent, delay the progression of AS and alleviate related clinical symptoms, they are associated with various adverse drug reactions, such as rhabdomyolysis (Ben et al., 2020), liver function impairment (Bellosta and Corsini, 2018), and an increased risk of diabetes (Michaeli et al., 2023). Moreover, interventional procedures like balloon angioplasty or stent implantation, while capable of reopening occluded arteries, are often accompanied by vascular remodeling and neointimal hyperplasia, ultimately leading to restenosis of the vascular lumen, and this remains a common occurrence (Bajeu et al., 2024). Hence, there is a need to identify more effective and safe drugs for the prevention and treatment of AS.

Throughout history, plant-based preparations have been widely used in medicine due to their lower toxicity and side effects. Increasingly, researchers have discovered safe, effective, and low-toxicity compounds with anti-AS properties in natural products, such as Ginkgo Biloba extract B (Ye et al., 2024), kaempferol (Chen et al., 2024), emodin (Wang et al., 2023), and paeoniflorin (Yu et al., 2022). Luteolin (3′,4′,5,7-tetrahydroxyflavone) is a naturally occurring tetrahydroxyflavone compound abundant in plants. In recent years, numerous studies have elucidated its potential anti-AS effects. Consequently, this review will comprehensively discuss the anti-AS effects of luteolin.

## 2 Luteolin

**FIGURE 1:**
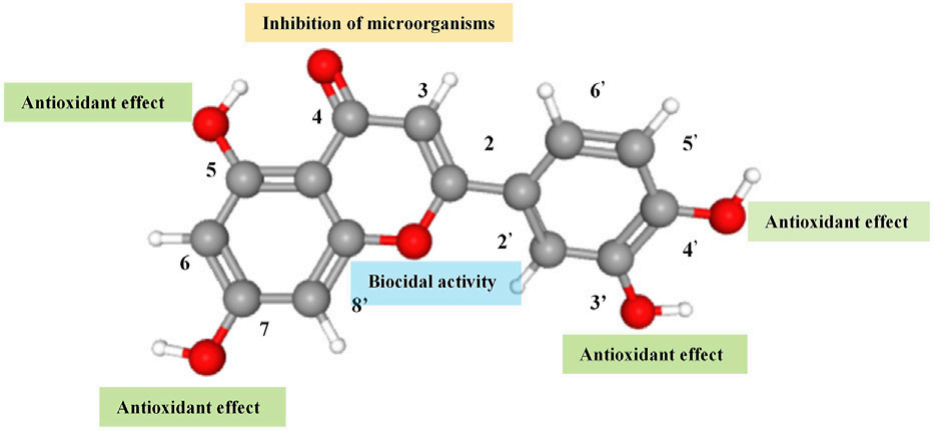
The molecular structure and function of the functional groups of luteolin.

Flavonoids are a class of benzopyrone derivatives widely present in the plant kingdom, characterised by their molecular structures containing phenolic and pyran rings. The basic structure of these compounds mostly consists of two benzene rings (A and B rings) tightly linked by a central three-carbon chain (C ring), forming a stable C6-C3-C6 skeleton. This configuration endows flavonoids with extreme chemical diversity and remarkable biological activity, demonstrating potential applications in various fields (Fusi et al., 2020). Luteolin, a common plant secondary metabolite and representative flavonoid, plays a crucial role in protecting plant cells from UV radiation or attracting pollinators and seed dispersers. Luteolin typically appears as yellow crystals, exhibits good thermal stability (Le Marchand, 2002), is slightly soluble in water but soluble in alkaline solutions and organic solvents such as ethanol and ether. Due to its colour, plants containing luteolin, like the reseda, have been used as dyes since 1000 BC. Luteolin was first isolated by the French chemist Michel Eugène Chevreul in 1829, but its correct structure was proposed by the British chemist Arthur George Perkin in 1896. Luteolin possesses a diphenylpropane structure (C6-C3-C6) with a molecular formula of C15H10O6 and a molecular weight of 286.24; its chemical structure is shown in Figure 1. The hydroxyl groups at the 5, 7, 3′, and 4′carbon positions, as well as the double bond between the 2 and 3 carbon positions, contribute to luteolin’s diverse pharmacological effects. Luteolin’s strong antioxidant activity is attributed to the hydroxyl groups at the aforementioned carbon positions; while the carbonyl oxygen at the 4 carbon position effectively inhibits microorganisms. Furthermore, the double bond between the 2 and 3 carbon positions provides luteolin with biocidal activity (Lin et al., 2008).

Luteolin is usually found in its glycoside form in vegetables and fruits such as celery, parsley, broccoli, onion leaves, carrots, peppers, cabbage, apple peels, and chrysanthemum, and it has varying contents (see Table 1), where one or more of its hydroxyl groups are substituted by sugar molecules (e.g., glucose, galactose, rhamnose) via glycosidic bonds. In recent years, numerous studies have revealed Luteolin’s significant role in maintaining human health, including anti-sepsis (Vajdi et al., 2023), anti-cancer (çetinkaya and Baran, 2023), liver protection (Yao et al., 2023), kidney protection (Diniz et al., 2022), anti-Parkinson’s (Siddique, 2021), influencing glycolipid metabolism (Wang et al., 2021), and anti-neuroinflammation (Cordaro et al., 2020). Numerous studies have also suggested Luteolin’s potential efficacy in anti-AS therapies. This article systematically elaborates on Luteolin’s pharmacological mechanisms against AS and its regulation of common AS risk factors. We believe this analysis will lay a foundation for Luteolin’s pharmacological research, clinical application, and product development in anti-AS therapies (In addition to the main text, we have also presented relevant information in a table format for easier reader access).

**TABLE 1:** The source and content of luteolin.

Category	Sources	Contents (mg/100 g)
Other plants	Raw dishcloth gourd (Luffa)	0.01 (fresh weight,FW)
Vegetables	Raw cabbage (*Brassica oleracea* var. capitata)	0.04 (FW)
Vegetables	Raw red cabbage (*Brassica oleracea* var. Capitata f. rubra)	0.06 (FW)
Vegetables	Raw lettuce iceberg (Lactuca sativa var. capitata)	0.06 (FW)
Vegetables	Raw cabbage Chinese (*Brassica pekinensis*)	0.06 (FW)
Vegetables	Raw cauliflower (*Brassica oleracea* var. botrytis)	0.08 (FW)
Other plants	Parsley (Petroselinum crispum)	0.14 (dry weight,DW)
Other plants	Raw chives (Allium schoenoprasum)	0.15 (FW)
Fruits	Raw sweet potato leaves (Ipomoea batatas L. folium)	0.20 (FW)
Other plants	Sage (Salvia officinalis)	0.20 (DW)
Other plants	Walnut (Juglans regia)	0.29 (DW)
Vegetables	(*Brassica oleracea* gemmifera)Raw brussels sprouts	0.34 (FW)
Vegetables	Raw beets (Beta vulgaris)	0.37 (FW)
Other plants	(Narrow-leaved Germander)Teucrium polium	0.48 (DW)
Vegetables	Raw red sweet peppers (Capsicum annuum L.)	0.63 (FW)
Vegetables	Raw green sweet peppers (Capsicum frutescens L. var. grossum Bailey)	0.69 (FW)
Vegetables	White radish (Raphanus sativus)	0.90 (DW)
Vegetables	French bean (Phaseolus vulgaris)	1.10 (DW)
Vegetables	Raw Spinach (Spinacia oleracea)	1.11 (FW)
Other plants	None(Teucrium chamaedrys)	1.20 (DW)
Vegetables	Raw parsley (Petroselinum crispum)	1.24 (FW)
Fruits	Raw mango fruit (Mangifera indica)	1.25 (FW)
Vegetables	Raw Kohlrabi (*Brassica oleracea* L. var. gongylodes L.)	1.30 (FW)
Vegetables	Raw celery (*Apium graveolens* L.)	1.31(FW)
Vegetables	Raw peppers, jalapeno (Capsicum annuum)	1.34 (FW)
Fruits	Raw lemons fruit (Citrus × limon (Linnaeus) Osbeck)	1.50 (FW)
Other plants	Rosemary (Rosmarinus officinalis)	1.60 (DW)
Medicinal herbs	Perilla leaves (Perilla frutescens)	1.68 (FW)
Other plants	Parsley (Petroselinum sativum)	2.10 (DW)
Other plants	Celery (*Apium graveolens*)	2.31 (FW)
Other plants	Spartium junceum (Spartium junceum L.)	2.50 (DW)
Vegetables	Limau purut leaves	3.05 (DW)
Other plants	Cumin (Cuminum cyminum)	3.10 (DW)
Vegetables	Green chili (Capsicum annuum)	3.30 (DW)
Other plants	Catnip (Nepeta cataria)	3.60 (DW)
Vegetables	Carrot (Rosmarinus officinalis)	3.75 (DW)
Other plants	Fresh rosemary (Rosmarinus officinalis)	4.00 (FW)
Other plants	Olive (Olea europaea)	4.00 (DW)
Vegetables	Raw pepper Serrano (Capsicum annuum)	4.14 (FW)
Vegetables	Raw green peppers (Peppers, capsicum, green, raw)	5.11 (FW)
Other plants	Broccoli (*Brassica oleracea* var. italica)	7.45 (DW)
Other plants	Local celery (*Apium graveolens* L.)	8.05 (DW)
Other plants	Asam gelugor (Asarum gelasinum)	10.75 (DW)
Vegetables	Peppermint fresh (Mentha × piperita)	11.33 (FW)
Other plants	Crepis incana	13.00 (extract, EXTR)
Other plants	Fresh sage (Salvia officinalis)	16.70 (FW)
Vegetables	Dried parsley (Petroselinum crispum)	19.75 (FW)
Fruits	Belimbi fruit (Averrhoa bilimbi)	20.20 (DW)
Medicinal herbs	Fresh Mexican oregano (Lippia graveolens)	25.10 (FW)
Vegetables	Raw Chinese celery (*Apium graveolens* L. var. dulce DC.)	34.87 (FW)
Other plants	common thyme (Thymus vulgaris)	36.00 (DW)
Vegetables	Radicchio (Cichorium intybus L.)	37.96 (FW)
Vegetables	Onion (Allium cepa)	39.10 (DW)
Other plants	Sweet Bay(Laurus nobilis)	39.34 (DW)
Other plants	Branched Asphodel (Asphodelus ramosus)	50.00 (DW)
Medicinal herbs	Thyme fresh (Thymus vulgaris)	51.00(FW)
Other plants	Clusii’s Peony (Paeonia clusii)	69.00 (DW)
Medicinal herbs	Juniper berries (Juniperus communis)	69.05 (FW)
Vegetables	Bird chili	103.50 (DW)
Medicinal herbs	Oregano (Origanum vulgare)	1,028.80 (FW)

## 3 Methods

Literature We conducted literature searches across PubMed, Web of Science, and Google Scholar databases without applying any search filters. The keywords we used included “luteolin”, “3′,4′,5,7-Tetrahydroxyflavone”, “Luteoline”, “3′,4′,5,7-Tetrahydroxy-Flavone”, “atheroscleroses”, “atherosclerosis”, “hypertension”, “high blood pressure”, “diabetes mellitus”, “hyperlipemia”, “pharmacological effect”, “pharmacological mechanism”, “pharmacokinetics”, “safety”, “toxicity” and “drug Interaction”, All the articles we included were published between 2004 and 2024. The aim of this review is to delve into the therapeutic role and mechanisms of luteolin in atherosclerosis. To that end, we thoroughly reviewed the titles, abstracts, and full texts of publications from the past 20 years, eliminating those that were irrelevant to our review topic. We then categorized the remaining articles based on their mechanism types. Additionally, we conducted a correlation search to find citations to relevant studies and review literature. Duplicates were carefully removed using both the automatic and manual duplicate detection features of Endnote 20, resulting in a final inclusion of 32 references.

## 4 Antiatherosclerotic effect of luteolin

**FIGURE 2:**
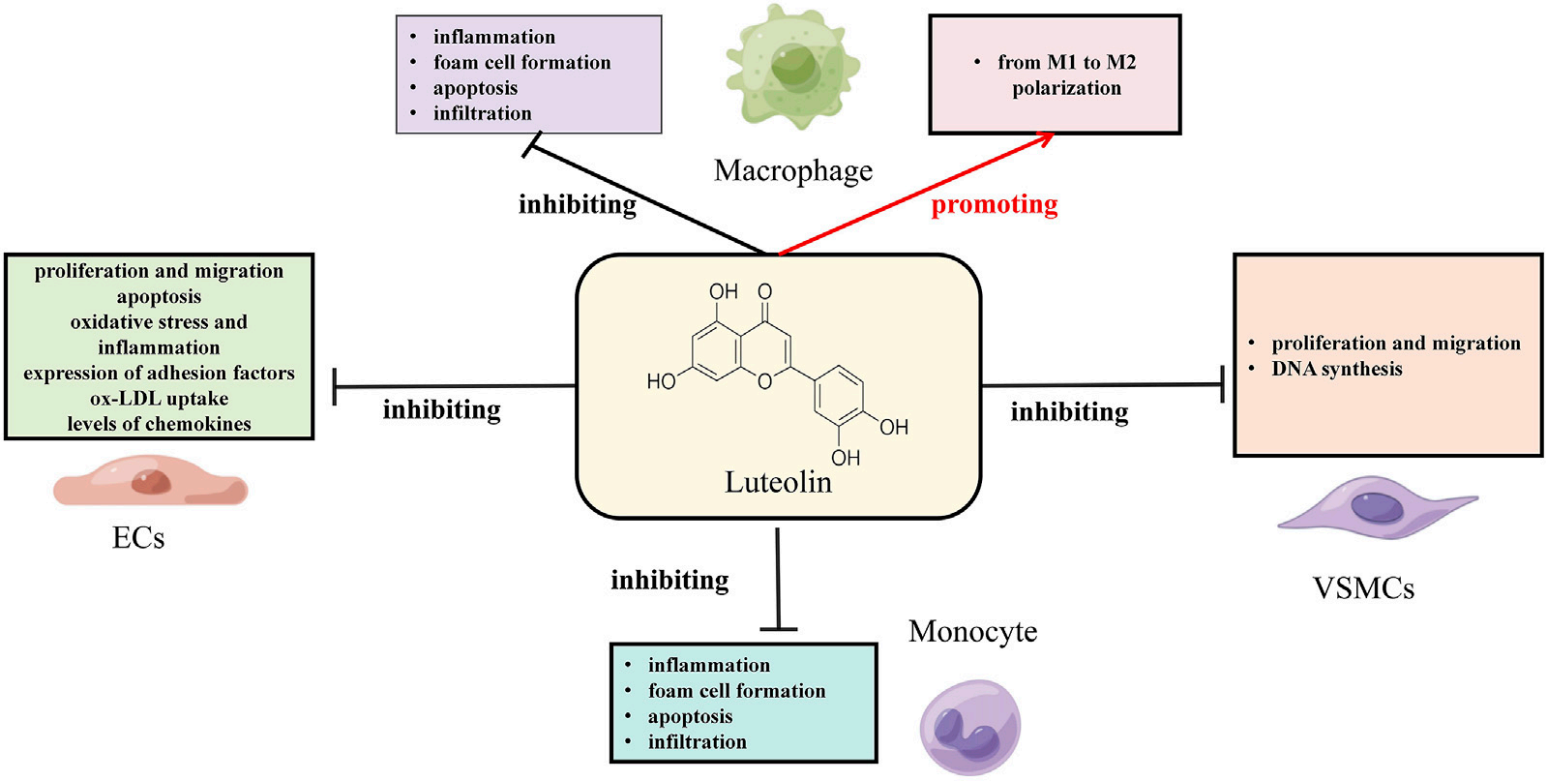
Effects of luteolin on various vascular cells under AS conditions.

AS is a vascular disease that involves multiple cell types, including endothelial cells, vascular smooth muscle cells (VSMCs), macrophages, and platelets. Dysfunction of these cells triggers a cascade of complex pathological reactions, manifested specifically as endothelial cell injury, persistent chronic inflammation, lipid metabolism imbalance, “excessive oxidative stress responses, abnormal proliferation and migration of VSMCs, and abnormal activation of platelets (Weber and Noels, 2011). The following discussion will delve into the various cell functions associated with AS that are affected by luteolin (see Figure 2; Table 2).

**TABLE 2:** Effects of luteolin on various vascular cells under AS conditions.

Cell types	Models and modeling methods	Research type	Dose and treatment schedule	Comparator drug	Targets	Effect	References
ECs	HUVECs; 1 μmol/L Ang Ⅱ for 12 h	*In vitro*	Luteolin; 12.5.25,50 μmol/L for 3 h,6 h,12 h,24 h	—	↓Src,-p-Src,↓p-Akt (308),↓p-Akt (473),-Akt in cells	Inhibiting endothelial cell proliferation and migration	Zhu et al. (2013)
	HUVECs; 100,150 μmol/L LPC for 18 h	*In vitro*	Luteolin; 1.10,100 μmol/L for 2 h	—	↓LDH,↓[Ca2+]i,↓calpains activity,↓caspase-3 activity in cells; ↓cytochrome C in the cytosol,↑cytochrome C in the mitochondrion	Inhibiting endothelial cell Apoptosis	Song et al. (2010)
	HUVECs; 50 ng/mL TNF-α for 24 h	*In vitro*	Luteolin; 6.25.12.5.25 μmol/L for 12 h	Quercetin (20 μM)	↓LDH,↑SOD,↑GSH、↓ROS,↓NOX4,↓p22phox,↓caspase-3,↓caspase-9,↑Bcl-2,↓ICAM-1,↓VCAM-1,↑IκB-α,↓p-p65,-p65,↓p-p38,-p38,↓p-ERK1/2,-ERK1/2	Reducing oxidative stress, inflammation and apoptosis	Xia et al. (2014)
	HUVECs; 10 ng/mL TNF-α for 6 h; THP-1 cells	*In vitro*	GTE (40,80,120,160μg/mL),Luteolin (5.10,20 μM),L7G (5.10,20 μM) for 2 h	Oleanolic acid (5.10,20 μM)	↓ICAM-1,↓VCAM-1,I↑κBα in cells	Inhibit the expression of adhesion factors of ECs and reduce the adhesion of monocytes	Hsuan et al. (2015)
	HUVECs; 0.1 mg/mL ox-LDL for 5 h; THP-1 cells	*in vitro*	Luteolin; 25 µM for 12 h	—	↓VCAM-1,↓E-selectin,↓LOX-1 in HUVECs	Inhibiting monocyte adhesion and ox-LDL uptake	Jeong et al. (2007)
	EA.hy926 cell; 10 ng/mL human recombinant TNF-α for 24 h; THP-1 cells and WEHI 78/24monocytes; C57BL/6 mice; TNF-α 25 μg/kg was injected intraperitonealy for 1week	*In vivo* and *in vitro*	Luteolin; 0.5µM–20 µM for 1 h; fed with AIN-93G rodent diet containing 0.6% Lu	—	↓MCP-1,↓VCAM-1,↓ICAM-1,↓IκBα,↓IKKβ in cells; ↓NF-κB p65(In the nucleus), ↓MCP-1/JE,↓CXCL1-KC,↓ICAM-1 in Plasma; ↓VCAM-1 in vascular tissue	Reducing the adhesion of monocytes to ECs; Reducing levels of chemokines and adhesion molecules in plasma and endothelial cells	Jia et al. (2015)
VSMCs	RASMCs; 50 ng/mL PDGF-BB for 24 h	*In vitro*	L7G; 0,5,20,50 μmol/L for 24 h	—	↓p-PLC-γ1,↓p-Akt,↓p-ERK1/2 in cells	Inhibiting vsmc proliferation and DNA synthesis	Kim et al. (2006)
	A7r5 and HASMC cell line	*In vitro*	Luteolin; 10.20,40 µM for 48 h	—	↓PCNA,↓Cyclin D1,↓MMP2,↓MMP9,↓p-TGFBR1(Ser165),-TGFBR1,-Smad2,-Smad3,↓p-Smad2(Ser 465/Ser 467),↓p-Smad3(Ser 423/Ser 425),-Bax,-Bcl-2	Inhibiting vsmc proliferation and migration	Wu Y. T. et al. (2018)
	Primary vascular smooth muscle cell (From SD rats)	*In vitro*	Luteolin; 25, 50 µM for 12 h	—	↓p-PDK1,↓p-Akt (308),↓p-Akt (473),-Akt,↓p-Src,-Src	Inhibiting vsmc proliferation and migration	Lang et al. (2012)
Macrophages	RAW264.7 cells; 100 ng/mL LPS for 6 h	*In vitro*	Luteolin; 2 μmol/L for 24 h	—	↓TNF-α,↓IL-6,↓IL-18,↓IL-1β,↑IL-10,↓ROS,↓NLRP3,↓ASC,↓caspase-1,↓iNOS,↑Arg-1 in cells	Inhibiting the activation of NLRP3 inflammasome, promote the polarization of macrophages to M2 phenotype, and play an anti-inflammatory role	Zhang et al. (2018)
	RAW264.7 cells; 50 μg/mL ox-LDL for 24 h	*In vitro*	Luteolin; 25 μmol/L for 24 h	—	↓Bax,↓Bak,↓cleaved caspase-9,↓cleaved caspase-3,↑Beclin-1,↑LC3-II/LC3-I,↓TC,↓CE in cells	Reducing foam cell formation and macrophage apoptosis by promoting autophagy	(Zhang et al., 2016)
	LDLR^−/−^ knockout mice with a C57BL/6 background; western-type diet for 14 weeks; THP-1 cells; 100 nM phorbol myristate acetate for 48 h,100 μg/mL ox-LDL for 5 h at 37°C	*In vivo* and *in vitro*	Luteolin; 100 mg/kg/d p.o.for 14 weeks; Lu; 0, 5, 10 and 20 µM for 24 h	—	↓CD68,↓CCL2,↓IL-6,↓TNF-α in the aortic root of mice; ↓TC,↓TG,↓LDL-c,-HDL-c in the serum of mice; ↓CD68,↓CCL2,↓IL-6,↓TNF-α,↓TC,↓TG,↑p-AMPK/AMPK,↑SIRT1 in cells	Inhibiting macrophage infiltration and inflammation to reduce arterial plaque formation and lipid accumulation	Li et al. (2018)
	Primary mouse peritoneal macrophages (From C57BL/6 mice); AngⅡ 1 μM for 12 h	*in vitro*	Luteolin; 25 µM for 12 h	—	↑Bcl-2,↑caspase-3,↓Bax,↓cleaved caspase-3,↓IL-6,↓TNF-α,↓iNOS,↓CD16/32,↑Dectin-1,↑IL-10,↑Arg-1,↑CD206,-AKT,↓p-AKT (308),↓p-AKT (473) in cells	Promoting the macrophages from M1 to M2 polarization to inhibit cell apoptosis	Jiang et al. (2018)
	MPM 50 μg/mL ox-LDL for 24 h; ApoE−/−mice HFD for12w	*In vivo* and *in vitro*	Luteolin; 2.5.5,10 μM for 12 h; 10 mg/kg/2days p.o.for 12 weeks	—	↓ICAM-1,↓VCAM-1,↓IL-6,↓TNF-α,↓p-STAT3,-STAT3 in cells; LDL-C,HDL-C,TCH,TG in serum; ↓ICAM-1,↓VCAM-1,↓IL-6,↓TNF-α,↓p-STAT3,-STAT3 in the aortic	Suppressing inflammation	Ding et al. (2019)
Monocyte	HUVECs and THP-1; TNF-a 10 ng/mL、ox-LDL 20 μg/mL for various time; THP-1 derived macrophages ox-LDL 20 μg/mLor PDGF-BB 10 mmol/L for various time	*in vitro*	Luteolin; 1,5,10,20 µM for 12 h	—	↓integrin β2,↓MMP-9,in THP-1 cells; ↑occludin,-PECAM-1, in HUVECs; ↓PDGF-BB,↓SR-A1,↓SR-B1 in THP-1 derived macrophages	Inhibition of monocyte-endothelial cell adhesion, monocyte migration, and foam cell formation	Kim et al. (2012)
	THP-1 cells; Add 100 nM TPA for 24 h	*in vitro*	Luteolin; 20 µM for 1 h	—	↓CD11 b,↓CD14,↓CD36,-MEK,↓p-MEK,-ERK,↓p-ERK,↓NOX2,↓P47phox,↓ROS in THP-1 cells	Inhibiting oxidative stress and inflammation	Makino et al. (2013)

### 4.1 Effects on endothelial cells

The Endothelial cells (ECs) play a crucial role in the acceleration and initiation of AS (Adawi et al., 2019; Higashi et al., 2009). ECs apoptosis is a key event in the progression of AS. Song et al. (2010) found that luteolin could reduce lysophosphatidylcholine (LPC)-induced apoptosis and lactate dehydrogenase (LDH) leakage in human umbilical vein endothelial cells (HUVECs) in a concentration-dependent manner. LPC stimulation leads to increased intracellular calcium ion concentrations, elevated calpain activity, altered cytochrome C levels (increased in the cytoplasm and decreased in the mitochondria), and increased caspase-3 activity, all of which are inhibited by luteolin. This suggests that luteolin can inhibit LPC-induced ECs apoptosis by blocking the calcium-dependent mitochondrial pathway. Plaque rupture is a significant contributor to cardiovascular events, and angiogenesis is believed to be a major cause of unstable AS plaque rupture (Moreno et al., 2006). The proliferation and migration of ECs are important for angiogenesis (Fosbrink et al., 2006; Folkman, 1995). Zhu et al. (2013) discovered that luteolin can reverse angiotensin II (Ang II)-induced migration and proliferation of ECs by downregulating the PI3K/Akt signaling pathway. Further mechanistic studies revealed that luteolin inhibits the upregulation of p-Src, p-Akt (308), and p-Akt (473) in ECs in a dose- and time-dependent manner. Oxidative stress and inflammation in ECs play pivotal roles in AS, influencing each other and jointly disrupting EC integrity and function, promoting foam cell formation and plaque progression, ultimately leading to the development of AS. Xia et al. (2014) found that luteolin alleviates TNF-α-induced oxidative stress and inflammation in HUVECs by inhibiting the Nox4/ROS/NF-κB and p38, ERK1/2 pathways. Dysfunctional ECs release pro-inflammatory cytokines, which activate and recruit monocytes, thereby promoting AS formation and development. Hsuan et al. (2015) reported that luteolin and luteolin-7-glucoside (lut-7-g) can attenuate the expression of intercellular adhesion molecule-1 (ICAM-1) and vascular cell adhesion molecule-1 (VCAM-1) in TNF-α-activated HUVECs and inhibit the adhesion of monocytes to these cells. Jeong et al. (2007) found that luteolin inhibits the adhesion of THP-1 cells to ox-LDL-stimulated HUVECs and reduces the expression of adhesion molecules VCAM-1, E-selectin mRNA, and protein. Additionally, luteolin inhibits the expression of lectin-like oxidized LDL receptor-1 (LOX-1) mRNA and protein in ox-LDL-stimulated HUVECs, reducing cellular uptake of ox-LDL, thereby inhibiting the production of adhesion molecules and blocking the adhesion of monocytes to ox-LDL-stimulated ECs, exerting anti-AS effects. Jia et al. (2015) reported that *in vitro* studies showed that luteolin pretreatment could reduce or even completely block the adhesion of THP-1 cells to EA. hy926 cells induced by human recombinant TNF-α. Further research revealed that luteolin pretreatment significantly reduced the expression of chemokine MCP-1 and adhesion molecules VCAM-1 and ICAM-1 mRNA in TNF-α-induced EA. hy926 cells. The activation of NF-κB plays a key role in the transcriptional regulation of chemokines and vascular adhesion molecules. The team further found that luteolin can inhibit the increased transcriptional activity of NF-κB, degradation of IκBα, nuclear translocation of NF-κB p65, and upregulation of IKKβ expression in TNF-α-induced EA. hy926 cells by inhibiting the NF-κB signaling pathway, thereby inhibiting EC inflammation. *In vivo* studies showed that dietary luteolin supplementation significantly prevented the adhesion of WEHI 78/24 monocytes to the vascular wall of C57BL/6 mice injected intraperitoneally with TNF-α and also reduced serum levels of CXCL1-KC (the mouse homologs of human MCP-1 and IL-8) and sICAM-1, as well as the expression of adhesion molecule VCAM-1 and the number of F4/80-positive monocyte-derived macrophages in the aorta of these mice. Therefore, the protective effect of luteolin on vascular endothelial inflammation may be mediated by inhibiting the IκBα/NF-κB pathway.

### 4.2 Effects on vascular smooth muscle cells

Vascular smooth muscle cells (VSMCs) are not only one of the main components of the arterial wall, but also play a crucial role in the pathological process of AS. The proliferation, migration, and phenotypic switching of VSMCs are important mechanisms underlying plaque formation, while their apoptosis is associated with plaque rupture. Platelet-derived growth factor (PDGF) is one of the key regulators of VSMC mitosis and is involved in the pathological proliferation of VSMCs in AS lesions. Kim et al. (2006) have found that luteolin 7-glucoside (L7G) can inhibit PDGF-BB-induced VSMC proliferation and DNA synthesis by blocking the phosphorylation of PLC-γ1, Akt, and Erk1/2, ultimately inhibiting VSMC proliferation. Wu G. et al. (2018) also discovered that luteolin may inhibit VSMC proliferation (rather than promoting apoptosis). Furthermore, their team found that luteolin can inhibit the phosphorylation of TGFBR1, Smad2, and Smad3 in VSMCs, as well as VSMC migration. Overexpression of TGFBR1 can partially reverse these effects of luteolin, suggesting that luteolin may inhibit VSMC proliferation and migration by inhibiting the TGFBR1 signaling pathway. Lang et al.'s research revealed that luteolin can significantly inhibit H2O2-induced VSMC proliferation and migration by downregulating the Akt and Src signaling pathways *in vitro* (Lang et al., 2012).

### 4.3 Effects on macrophages

As one of the primary and most widely distributed inflammatory cells, macrophages play a pivotal role in AS They contribute to disease progression by phagocytosing lipids to form foam cells, participating in plaque formation and instability, and secreting inflammatory factors. Macrophage polarization, the process where macrophages differentiate into functionally distinct M1 (pro-inflammatory) or M2 (anti-inflammatory) subtypes under specific microenvironmental stimuli, is closely associated with AS and significantly influences its progression. Zhang et al. (2018) found that luteolin promotes the transition of RAW264.7 cells from an M1 phenotype to an M2 phenotype (manifested by increased expression of Arg-1 and production of IL-10, decreased expression of iNOS and reduced production of TNF-α and IL-6) after lipopolysaccharide (LPS) stimulation. Concurrently, luteolin inhibits the production of reactive oxygen species (ROS), activation of the NLRP3 protein, and the release of pro-inflammatory cytokines (IL-1β, IL-18). This suggests that luteolin may exert anti-inflammatory and anti-AS effects by inhibiting NLRP3 inflammasome activation and promoting M2 macrophage polarization.

The formation and apoptosis of macrophage-derived foam cells are considered central to the development of AS (Zhang et al., 2018; Yuan et al., 2012; Tabas, 2009). Foam cell formation marks the early stages of AS, while their apoptosis further exacerbates plaque instability and lesion progression. Zhang et al. (2016) discovered that luteolin reduces the accumulation of lipid droplets in RAW264.7 cells treated with oxidized low-density lipoprotein (ox-LDL) and reverses elevations in total cholesterol (TC), esterified cholesterol (CE) levels, and ABCA1 expression. Furthermore, the team found that ox-LDL treatment increases apoptosis and the expression of apoptosis-related proteins (Bax, Bak, cleaved caspase-9, cleaved caspase-3) in RAW264.7 cells, whereas luteolin significantly inhibits macrophage apoptosis. Meanwhile, luteolin treatment of foam cells induces the formation of MDC-labeled autophagosomes and increases the ratio of Beclin-1 and C3-II/LC3-I. The addition of the autophagy inhibitor 3-MA reverses these effects of luteolin on macrophages, indicating that luteolin promotes cholesterol efflux, reduces foam cell formation, and inhibits macrophage apoptosis by activating autophagy, thereby exerting anti-AS effects. Jiang et al. (2018) found that luteolin reverses the increased apoptosis rate, Bax, and cleaved caspase-3 levels, as well as the decreased Bcl-2 and caspase-3 levels, in mouse peritoneal macrophages stimulated by angiotensin II (Ang II). Additionally, luteolin reduces the expression of M1 macrophage markers (IL-6, TNF-α, iNOS, CD16/32) and increases the expression of M2 macrophage markers (Dectin-1, IL-10, Arg-1, CD206). Further mechanistic studies revealed that luteolin reverses the upregulation of Akt phosphorylation at residues 308 and 473, with effects similar to those of the PI3K/Akt pathway-specific inhibitor LY294002. The combination of luteolin and LY294002 shows no significant difference compared to their individual use, indicating that luteolin may promote macrophage polarization (from M1 to M2) by inhibiting the PI3K/Akt signal, ultimately inhibiting Ang II-stimulated macrophage apoptosis.

The infiltration of macrophages and the subsequent inflammatory response are fundamental pathological bases for the formation and development of AS. Li et al. (2018) found that luteolin prevents aortic lipid accumulation and atherosclerotic plaque formation in LDLR −/− mice induced by a Western diet. It also reverses elevations in total cholesterol, triglycerides, and LDL-c levels in mouse plasma, as well as levels of the macrophage marker CD68, macrophage chemoattractant protein-2 (CCL2), IL-6, and TNF-α in the aortic root. Further *in vitro* studies revealed that luteolin dose-dependently reduces cholesterol content and levels of CCL2, IL-6, and TNF-α in THP-1 cells induced by ox-LDL. Additional mechanistic studies showed that luteolin increases levels of p-AMPK/AMPK and SIRT1 in THP-1 cells induced by ox-LDL, suggesting that luteolin may reduce arterial plaque formation and lipid accumulation by activating the AMPK/SIRT1 signaling pathway to inhibit macrophage infiltration and inflammation. Ding et al. (2019) found that, without affecting blood lipid levels, luteolin reverses the significant elevations in inflammatory factors and adhesion molecules, including ICAM-1, VCAM-1, IL-6, and TNF-α, in the aortas of ApoE^−/−^ mice. It also reduces macrophage infiltration in the aortic root, significantly decreases STAT3 phosphorylation levels, and alleviates AS pathological damage in HFD-induced ApoE^−/−^ mice. Further *in vitro* and *in vivo* studies revealed that L、luteolin similarly reduces ICAM-1, VCAM-1, IL-6, TNF-α, and p-STAT3 levels in oxLDL-induced primary mouse peritoneal macrophages (MPMs). After introducing STAT3 siRNA into macrophages, oxLDL does not increase these adhesion molecules and pro-inflammatory factors in STAT3 gene-knockout MPMs. Furthermore, luteolin does not further reduce the expression of these adhesion molecules and pro-inflammatory factors induced by ox-LDL.

### 4.4 Effects on monocytes

Monocytes play a pivotal role in the initiation and progression of AS. Their recruitment, adhesion, differentiation into macrophages, and the subsequent inflammatory response contribute to the formation, progression, and instability of AS plaques. Studies by Kim et al. (2012) have revealed that luteolin can inhibit the increase of integrin-β2 in THP-1 cells induced by ox-LDL, reduce the adhesion of THP-1 cells and TNF-α-stimulated HUVECs, and reverse the enhanced expression of MMP-9 and cellular migration ability in TNF-α-stimulated THP-1 cells. Furthermore, the research also found that luteolin can reverse the downregulation of Occludin in TNF-α-stimulated HUVECs and the increased expression of SR-A and SR-B1, which are related to the secretion of PDGF-BB, in THP-1-derived macrophages induced by ox-LDL. luteolin also inhibits the uptake of ox-LDL by macrophages and the formation of foam cells. Junya Makino’s study demonstrated that luteolin can reverse the expression of CD11b, CD14, and CD34 during THP-1 differentiation induced by 12-O-Tetradecanoylphorbol 13-acetate (TPA), while inhibiting cellular adhesion. Additionally, luteolin completely blocks TPA-induced ROS generation and TPA-triggered NOX2 expression and membrane translocation of p47phox, indicating that luteolin inhibits NOX2 activation, which subsequently prevents TPA-triggered CD family activation and reduces THP-1 cell adhesion (Makino et al., 2013).

## 5 Effect on risk factors associated with AS

**FIGURE 3:**
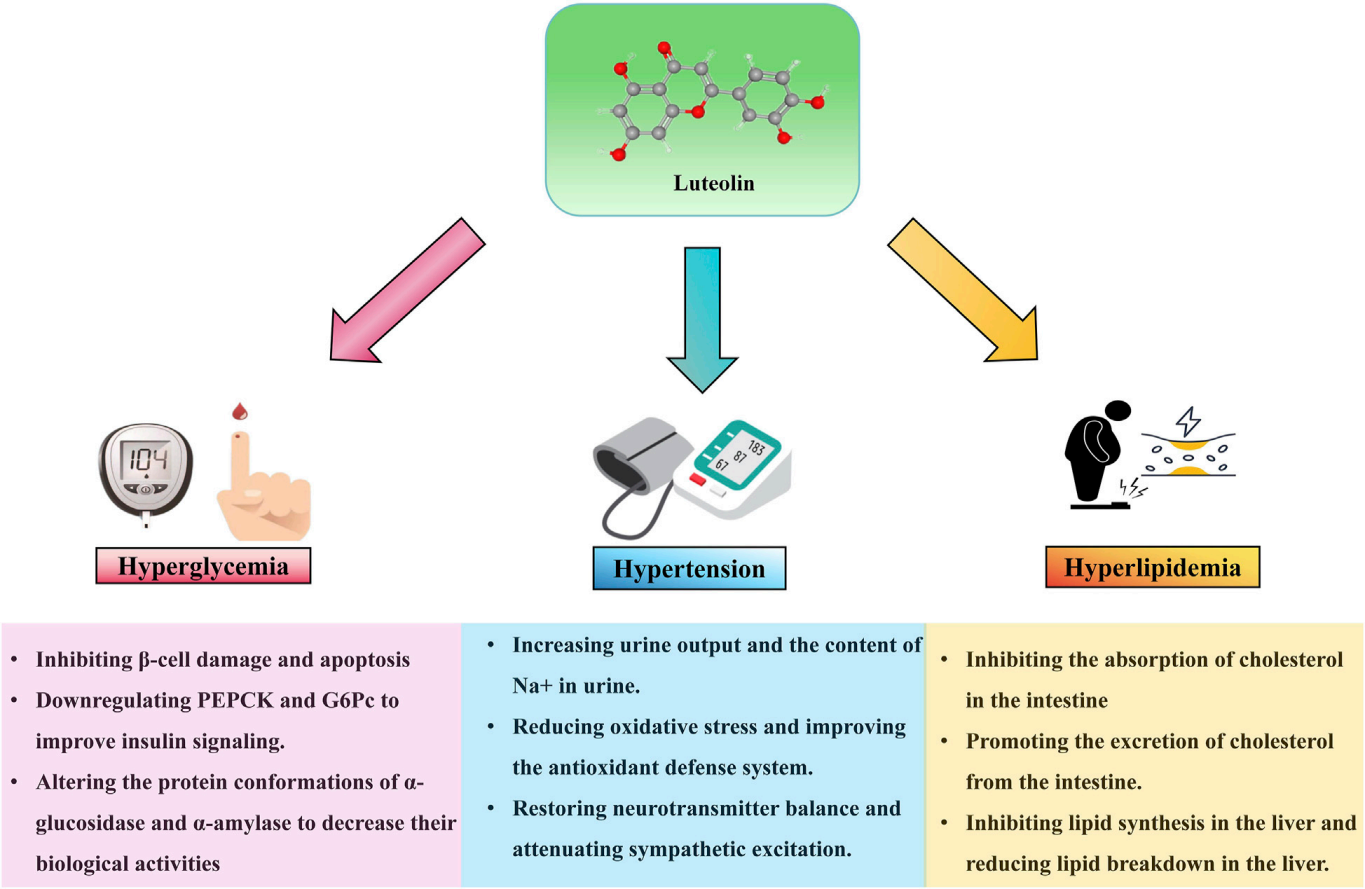
Effect of luteolin on risk factors associated with AS 9.

Multiple risk factors are closely associated with the occurrence and development of AS. These risk factors include, but are not limited to, diabetes, hyperlipidemia, hypertension, and others, which individually or synergistically contribute to the pathological progression of AS. The following sections will discuss the impact of luteolin on the risk factors related to AS (see Figure 3; Table 3).

**TABLE 3:** Effect of luteolin on risk factors associated with AS.

Risk factors	Models and modeling methods	Research type	Dose and treatment schedule	Comparator drug	Targets	Effect	References
Hyperlipidemia	Caco-2cells; 1 μmol/L Ang Ⅱ for 12 h Wistar rats were fed with HC at 5 mL/kg body weight	*In vivo* and *in vitro*	Luteolin; 100 mM for 1h; 20 mM p.o.for 10 d	Ezetimibe (20 mM),quercetin (100 mM)	↓NPC1L1 in cells	Inhibiting intestinal cholesterol absorption	Nekohashi et al. (2014)
	C57BL/6 mice; Feeding with a high-fat diet	*In vivo*	Luteolin; Incorporating into the feed at proportions of 0.005% and 0.025%. For 8weeks	—	↓NPC1L1,↓Srebf-2,↑abcg-5/8 in the small intestine; ↓HMGCR, ↓Srebp-2,-SREBP-2,-Ldlr,-Cyp7a1,↑p-AMPK,-AMPK, in the liver	Inhibiting the absorption of cholesterol in the intestine and promoting the excretion of cholesterol from the intestine	Wong et al. (2017)
	Liver cancer HepG2 cells and non-cancer WRL cells	*In vitro*	Luteolin; 0.1,1,5,10.25 μM for 24 h	—	↓FULL-length SREBP-2,↓C-terminal SREBP-2,↓N-terminal SREBP-2,↓HMGCR,↑p-AMPK,-t-AMPK,↑AMP/ATP in cells	Inhibits the synthesis of cholesterol	Wong et al. (2015)
	SD rats; Feeding with a high-fat diet for 4weeks	*In vivo*	Luteolin; 5 mg/mL p.o. For 6weeks	Simvastatin (10 mg/kg)	↓HMG-CoA,↓DGAT,↑FAβO,↑CYP7A1,↑HL,↑GSH-Px,↑CAT,↑SOD	Regulating antioxidant levels and enzyme activities related to lipid metabolism in the liver	Sun et al. (2021)
hypertension	Female wistar normotensive rats (NTR) and spontaneously hypertensive rats (SHR)	*In vivo*	Luteolin; 0.3, 1 or 3 mg/kg p.o	Hydrochlorothiazide (10 mg/kg),amiloride (3 mg/kg)	↓Ca2+,-k+,↓Na+ in Urine	It has natriuretic and diuretic effects	Boeing et al. (2017)
	Wistar albino rats; 300 mg/L NaF in Drinking water	*In vivo*	Luteolin; 100 and 200 mg/kg p.o. For 9 d	—	MDA↑MPO,↑AOPPs,↑ XO,↑NO bioavailability in serum	Reducing oxidative stress and improving antioxidant defense system	Oyagbemi et al. (2018)
	Wistar Kyoto (WKY) rats and SHR	*In vivo*	Luteolin; 20 µg/0.11 µL, volume: 0.11 μL/h,Bilateral PVNS were injected	—	↑SOD-1, ↑NOX2, ↓IL1β,↓IL6 ↓p-PIKKβ, ↓NFκB, ↓tyrosine hydroxylase (TH),↑ 67-kDa isoform of glutamate decarboxylase (GAD67),↓ROS,↓p-PI3K/PI3K,↓p-Akt/Akt in the PVN.↓NE,↓EPI in serum	inhibiting oxidative stress and inflammatory components, as well as restoring neurotransmitter balance	(Gao et al., 2023a)
Hyperglycemia	The INS-1E (rat insulinoma) cell line; 0.4 mM or 0.25 mM PA for 24 h	*In vitro*	Luteolin; 2.5.5,10 μM for 24 h	—	↓DRAK2,↓PARP,↓Cleaved-caspase 3,↓Cleaved-caspase 9,↑LC3-II/LC3-I,↓p62,↑ULK1,ROS in cells	Alleviating oxidative stress and promote β-cell autophagy, thereby improving β-cell function	Han et al. (2023)
	INS-1cells; Thapsigargin (THAP) for 24 h	*In vitro*	Luteolin; 2.5.5,10 μM for 24 h	—	↑Cytoplasmic Ca2+,↓sXBP-1,↓ATF-4, ↓BIP,↓CHOP,↓TXNIP,↓Cleaved-caspase 3,↓TNF,↓CXCL1,↓HNF1α,↓ANKS4b,↓HNF4αin cells in cells	Targeting HNF4α and inhibiting the expression of its downstream genes can alleviate the ER stress state of β cells	Wu et al. (2020)

### 5.1 Regulation of lipid metabolism

Hypercholesterolemia is one of the risk factors for AS. The balance of cholesterol in the human body is primarily maintained through intestinal absorption, endogenous biosynthesis, and biliary/intestinal excretion, with Niemann-Pick C1-like 1 (NPC1L1) on the brush border of the small intestine mediating cholesterol uptake in the gut. Ezetimibe, a drug that has been widely used, is an effective NPC1L1 inhibitor. Nekohashi et al. (2014) have found that luteolin can reduce high blood cholesterol levels by inhibiting NPC1L1-mediated intestinal cholesterol absorption. Wong et al. (2017) reported that luteolin not only downregulates the gene level of the transcription factor Srebf-2 in the intestine, which subsequently downregulates NPC1L1 to ultimately inhibit cholesterol absorption, but also increases the expression of abcg-5/8 in the mouse intestine, thereby promoting cholesterol excretion. Hydroxymethylglutaryl-CoA reductase (HMGCR) is responsible for cholesterol synthesis, and inhibitors of this enzyme (statins) have been clinically used to control serum cholesterol. Steroid regulatory element-binding protein (SREBP)-2 is a key transcription factor in cholesterol metabolism, and HMGCR is a target gene of SREBP-2. Inhibiting SREBP-2 activity may reduce HMGCR expression, thereby regulating cholesterol levels. Another study by Wong et al. (2015) revealed that luteolin can activate AMPK in hepatocyte lines WRL and HEPG2, blocking the post-translational processing of SREBP-2 protein and inhibiting its nuclear translocation, ultimately reducing the transcription level of HMGCR. Sun et al.'s research found that luteolin may increase the expression of antioxidant enzymes (GSH-Px, CAT, SOD) and enzymes related to lipid catabolism (FAβO, CyP7A1, HL) in rat livers, while decreasing the expression of lipid peroxidation products (MDA) and enzymes related to lipid synthesis (Fas, HMG-CoA, DGAT). This leads to an increase in body weight, a decrease in total cholesterol (TC), triglycerides (TG), and low-density lipoprotein cholesterol (LDL-C) levels, an elevation of blood HDL-C levels, a reduction in liver steatosis, and an improvement in liver function in rats (Sun et al., 2021).

### 5.2 Effect on hypertension

Hypertension is a significant risk factor in the formation and progression of AS, as it can accelerate this process by increasing pressure on the vascular walls, promoting endothelial damage, and facilitating lipid deposition. Numerous studies have elucidated the therapeutic effects of luteolin on hypertension. Boeing et al. (2017) discovered that luteolin exhibits diuretic properties, significantly increasing urine output and Na+ content in the urine of NTR and SHR rats without altering K+ and Cl− excretion. Further investigation revealed that these effects of luteolin could be markedly inhibited by atropine (a muscarinic receptor antagonist), indicating that luteolin may exert its diuretic and natriuretic effects by influencing muscarinic receptors. A study by Oyagbemi et al. (2018) found that luteolin could reverse the significant elevations in systolic blood pressure (SBP), diastolic blood pressure (DBP), and mean arterial pressure (MA) induced by sodium fluoride (NaF). Further mechanistic studies showed that luteolin could reverse the NaF-induced decreases in serum NO bioavailability and reduced glutathione (GSH) levels in rats, while also reducing oxidative stress-related indicators such as malondialdehyde (MDA) content, H2O2 production, and advanced oxidation protein products (AOPPs), as well as inhibiting the increased activity or content of myeloperoxidase (MPO), xanthine oxidoreductase (XO), and protein carbonyl (PCO). These results suggest that luteolin may exert its antihypertensive effects by reducing oxidative stress and improving the antioxidant defense system. Gao et al. (2023) found that injection of luteolin into the paraventricular nucleus (PVN) of the hypothalamus significantly reduced the mean arterial pressure (MAP), heart rate (HR), and circulating plasma levels of noradrenaline (NE) and adrenaline (EPI) in spontaneously hypertensive rats (SHRs). Additionally, the expression and activity of phosphatidylinositol 3-kinase (p-PI3K) and phosphorylated protein kinase-B (p-AKT), as well as the levels of reactive oxygen species (ROS) and NAD(P)H oxidase subunits (NOX2, NOX4), were decreased in the SHR PVN. Furthermore, the expression of inflammatory cytokines and the activity of nuclear factor-κB (NF-κB) p65 were also reduced in the SHR PVN. Moreover, immunofluorescence results indicated that luteolin injection decreased the expression of tyrosine hydroxylase (TH) and increased the expression of superoxide dismutase (SOD1) and the 67-kDa isoform of glutamic acid decarboxylase (GAD67), suggesting that luteolin may inhibit oxidative stress and inflammatory components, restore neurotransmitter balance, attenuate sympathetic excitation, and lower hypertension by activating the PI3K/AKT and NF-κB signaling pathways.

### 5.3 Effects on glucose metabolism

The high-sugar environment in the blood can damage vascular endothelial cells, making them more susceptible to injury and attracting inflammatory cells and lipid deposition, thereby further promoting the formation of AS plaques. Increased apoptosis and dysfunction of pancreatic β-cells are important causes of elevated blood glucose levels in diabetic patients. Numerous studies have elucidated the protective effects of luteolin on pancreatic islet cells. Han et al. (2023) found that luteolin can maintain β-cell function by inhibiting death-associated protein kinase-related apoptosis-inducing kinase-2 (Drak2), effectively alleviating palmitate (PA)-induced primary islet cell damage and β-cell apoptosis. Wu et al. (2020) discovered that luteolin targets the HNF4α/Asnk4b pathway to relieve endoplasmic reticulum stress-mediated β-cell death, which leads to insulin secretion disorders and a reduction in β-cell number. Enzymes related to glucose metabolism, such as hexokinase and phosphofructokinase, play crucial roles in maintaining blood glucose homeostasis and energy supply by regulating glucose absorption, conversion, and oxidative breakdown. Many studies have described the regulatory effects of luteolin on enzymes related to glucose metabolism. Bumke-Vogt et al. (2014) found that luteolin can also downregulate the mRNA of gluconeogenic enzymes phosphoenolpyruvate carboxykinase (PEPCK) and glucose-6-phosphatase (G6Pc), thereby inducing FOXO1 translocation, improving insulin signalling, and exerting hypoglycaemic effects. α-Glucosidase is vital for maintaining blood glucose homeostasis as it participates in the breakdown and absorption of carbohydrates, influencing postprandial blood glucose levels. Research by Djeujo et al. (2022) indicates that luteolin primarily binds to α-glucosidase through van der Waals contacts and hydrogen bonds, altering its protein conformation and ultimately reducing its biological activity. α-Amylase breaks down starch into glucose, directly affecting blood glucose levels, and plays a key role in maintaining blood glucose stability. Zhao et al.'s research reveals that luteolin binds to porcine pancreatic α-amylase (ppa) through hydrogen bonds and hydrophobic interactions, causing changes in its secondary structure, thereby reducing enzyme activity and effectively slowing down the digestion of starch and the rise in blood glucose levels (Zhao et al., 2021).

## 6 Safety, side effects, and drug interactions

The safety of natural products is of great concern to both clinicians and patients. In recent years, many scholars have investigated the toxicological effects of luteolin. In the *in vitro* toxicity study, the investigators used human retinal microvascular endothelial cells (HRMECs) and found that luteolin at 0–10 μM had no adverse effect on the cells, while luteolin at 100 μM caused a significant reduction in cell viability (Park et al., 2012). In another experiment, treatment of wild-type human lymphoblastoid TK6 cells with 2.5 μM luteolin produced cytotoxic effects within 24 h. The team also found that luteolin was genotoxic to TK6 cells, as indicated by a significant concentration dependent increase in the percentage of comet tail DNA after 4 h of luteolin treatment. Additionally luteolin larger than 5 μM for 24 h significantly increased γH2A.X protein level and percentage of micronuclei (%MN) (Li et al., 2021). *In vivo* toxicity studies showed that luteolin had an LD50 of 460 mg/kg in Male Institute of Cancer Research (ICR) mice, and 200 mg/kg luteolin caused liver and kidney toxicity in mice. No significant hepatorenal toxicity was observed in mice treated with luteolin at concentrations of 100 mg/kg and below (Xiong et al., 2017). In addition, the oral median lethal dose (LD (50)) of luteolin in mice and rats was higher than 2,500 mg/kg and 5,000 mg/kg, respectively (Kanai et al., 2016). Therefore, the above studies indicate that luteolin has a good safety profile.

Drug Interaction (DI) refers to the combined effects of two or more drugs taken at the same time or in a certain period of time. This effect may be manifested as a strengthening of the efficacy of the drug or a reduction of side effects, or it may lead to a weakening of the efficacy of the drug or the appearance of unwarranted toxic side effects. Many studies have described the interaction between luteolin and other drugs. Rakariyatham et al. (2018) found that both luteolin and sulforaphane inhibited LPS-induced nitric oxide (NO) production in macrophages in a dose-dependent manner. Compared with the single treatment, the combined treatment had a better inhibitory effect on the production of NO, the expression level of pro-inflammatory proteins in the NF-κB pathway, the activation level of STAT3, and the expression of its downstream inflammatory proteins such as iNOS, COX-2, IL-6, IL-1β. In addition, the combined treatment further reduced the ROS level and increased the expression of antioxidant proteins Nrf2 and HO-1 in the cells. Yan et al. (2019) found that the combination of metformin and luteolin could better improve the activity and expression of antioxidant enzymes, inhibit inflammatory response and reduce cell apoptosis than the single application, indicating that the combination of metformin and luteolin may have great potential and synergistic effect on ccl4-induced hepatotoxicity. Zhang et al. (2019) found that the combined inhibition of curcumin and luteolin on TNF-α-induced monocyte adhesion to ECs and MCP-1 and VCAM-1 expression was achieved by inhibiting the translocation of NF-κB to the nucleus. However, few studies have described the interaction between luteolin and commonly used anti-AS drugs (such as statins and antiplatelet drugs), which requires further investigation by future researchers through experimental and clinical studies.

## 7 Conclusion and prospects

AS is the most common pathophysiological mechanism in coronary atherosclerotic heart disease, cerebrovascular disease, and peripheral artery disease. These diseases pose a serious threat to public health worldwide due to their high morbidity and mortality. Based on the summary of the above, it is not difficult to find that luteolin has shown a wide range of potential in the prevention and treatment of AS, and its mechanism covers a variety of cell types, targets and signaling pathways. In addition, luteolin can also effectively intervene the risk factors closely related to AS, such as hyperglycemia, hypertension and dyslipidemia, so as to have an indirect positive effect on AS. Based on these findings, there are good reasons to believe that luteolin may be a highly promising therapeutic agent for AS. However, a number of challenges still need to be overcome to move luteolin from the laboratory to clinical use: (1) more comprehensive safety assessments are needed: Although luteolin is present in a variety of vegetables and fruits and is also a dietary supplement, its toxicity is low and its safety is good both *in vivo* and *in vitro* studies. However, in order to ensure its wide applicability in different organisms, we still need to conduct toxicological studies on a variety of animals, including dogs, rabbits, monkeys, etc., to achieve a comprehensive assessment of the safety of luteolin. (2) Inadequacy of clinical research evidence: Current studies mostly focus on preclinical studies, and clinical data are still insufficient. We searched the NIH clinicaltrials database (clinicaltrials.gov) and did not find any clinicaltrials using luteolin as an active ingredient in the treatment of AS or ASCVD. Large-scale randomized, placebo-controlled, double-blind clinical trials are urgently needed to evaluate the efficacy and safety of luteolin and determine the optimal dose, which will help promote its development as a functional food and drug. (3) The cellular level involved in the study needs to be expanded: Current studies mainly focus on endothelial cells, macrophages, smooth muscle cells and monocytes in the pathological process of AS. The effects and mechanisms of luteolin on T/B lymphocytes, mesenchymal stem cells, endothelial progenitor cells, red blood cells, mast cells and dendritic cells in the state of AS need to be further explored and improved. (4) Bioavailability needs to be improved: the low water solubility of luteolin (50.6 μg/mL) limits its bioavailability, thereby affecting its clinical application. In recent years, researchers have explored a variety of strategies to improve the water solubility, bioavailability, and efficacy of luteolin for the treatment of different diseases, such as forming complexes with cyclodextrins (Liu et al., 2013), phospholipids (Khan et al., 2016; Xu et al., 2009), and encapsulation using nanotechnology such as liposomes (Wu G. et al., 2018; Li et al., 2016), micelles (Zheng et al., 2017), nanoparticles (Majumdar et al., 2014), and microemulsion systems (Miyashita et al., 2022). Although these methods have made progress in improving the bioavailability of luteolin, the exploration of luteolin delivery systems in the treatment of strong AS is still limited. It is necessary to further evaluate the appropriate delivery vehicles to make full use of luteolin’s good anti-atherosclerotic effect. (5) Specific targets need to be identified: The direct targets of luteolin have not been identified in current studies, and the pathways identified in previous studies may only be part of the downstream signaling pathways after luteolin acts on specific molecular targets. Therefore, we suggest using systems biology methods to study the specific molecular targets of luteolin. (6) There are certain deficiencies in the research design. Through our summary, we found that most studies lacked positive drug controls, which hinders our understanding of the efficacy and safety of luteolin in treating atherosclerosis (AS). Currently, it remains unclear whether luteolin’s therapeutic effects differ from those of commonly used AS medications (e.g., statins) or known protective natural products (e.g., tanshinone IIA, resveratrol, curcumin). Future studies should incorporate such comparisons to validate luteolin’s clinical potential. (7) *In vivo* evidence is lacking: Many studies are currently limited to *in vitro* experiments, failing to capture the complexity of atherosclerosis *in vivo*. Future research should prioritize *in vivo* studies to fully understand luteolin’s effects on atherosclerosis.

In conclusion, although luteolin still faces many challenges in its translation into clinical applications, we firmly believe that with further research and continuous technological progress, luteolin is expected to become an important candidate for the prevention and treatment of AS. In the future, we look forward to more clinical research results on Luteolin to verify its safety and efficacy.
